# Further Nominations

**Published:** 1867-06

**Authors:** 


### FURTHER NOMINATIONS.

The Committee on Nominations made the following additional nominations:—

*On Prize Essays*—Drs. Charles Woodward, W. W. Dawson, S. B. Stevens, Robert Bartholow, P. S. Connor.

*Committee of Arrangements*—Drs. Grafton Tyler, Wm. P. Johnson, F. Howard, Wm. Murburry, Lewis Mackall, T. F. Mowry, J. M. Toner, of Washington, D.C.

*Assistant-Secretary*—J. W. H. Lovejoy, of Washington, D.C.

Dr. Samuel Willey was added to the Committee on Necrology, for Minnesota; also, Dr. S. M. Welch, of Texas.

